# Mitochondrial genome of the silky shark *Carcharhinus falciformis* from the British Indian Ocean Territory Marine Protected Area

**DOI:** 10.1080/23802359.2020.1775147

**Published:** 2020-06-11

**Authors:** Shaili Johri, Taylor K. Chapple, Elizabeth A. Dinsdale, Robert Schallert, Barbara A. Block

**Affiliations:** a Hopkins Marine Station, Stanford University, Pacific Grove, CA, USA; b Department of Biology, San Diego State University, San Diego, CA, USA; c Coastal Oregon Marine Experiment Station, Oregon State University, Newport, OR, USA

**Keywords:** Conservation, Chagos, CITES, fin-trade, genomics

## Abstract

We present the first mitochondrial genome of *Carcharhinus falciformis* from the Chagos Archipelago in the British Indian Ocean Territory (BIOT) Marine Protected Area (MPA). The mitochondrial genome of *C. falciformis* is 16,701 bp in length and consists of 13 protein-coding genes, 22 tRNA genes, 2 rRNA genes, and a non-coding control region (D-loop). GC content was at 40.1%. The control region was 1063 bp in length. The complete mitogenome sequence of *C. falciformis* from the BIOT MPA will enable improved conservation measures of the CITES listed species through studies of species distribution, population abundance, fishing pressure and wildlife trade.

The silky shark, *Carcharhinus falciformis* is a Vulnerable IUCN redlist and CITES listed species (Rigby et al. 2017). The species has a circumglobal distribution in tropical waters (Rigby et al. 2017). It is caught as targeted or incidental catch and is traded heavily for its meat and fins, with some regulations in place on catch and trade (Rigby et al. 2017). We sequenced the whole mitochondrial genome of the species sampled from the Chagos archipelago in the BIOT MPA to enable accurate identification of the species and stock population through molecular taxonomy. The *Carcharhinus falciformis* specimen was collected in the BIOT MPA on March 17, 2018. (Latitude: 07.1382°, Longitude: 072.1967°).

Fin tissue of a female specimen was stored in 70% ethanol post collection at the Hopkins Marine Station, Stanford University (Sample Accession # 020002232313). DNA extraction and sequencing was performed following Johri et al. (2019). Approximately 66 Fast5 sequencing files were converted to FASTQ files using the basecaller Guppy 3.3.1 (Oxford Nanopore Technologies) on a GPU interface. A total of 215,601 sequence reads were obtained with a length range of up to 50,000 bp. Reads were trimmed and mapped to mitogenomes from the genus Carcharhinus as described in Johri et al. 2019, resulting in a contig of 66 reads. The contig consensus sequence was annotated using orthologous loci following Johri et al. (2020).

To assess the phylogenetic position of *C. falciformis*, gene trees were constructed using complete mitochondrial genomes from six families within the order Carcharhiniformes and using Hexanchiformes as outgroup. Phylogenies were determined in Bayesian inference frameworks (Huelsenbeck and Ronquist 2001; Edgar 2004; Ronquist et al. 2012) following Johri et al. (2019, 2020). Bayesian trees were estimated using the GTR substitution model, gamma rate variations with 4 gamma categories, chain length 110,000, burn-in length 100,000 and subsampling frequency 200. *Carcharhinus falciformis* was nested within Carcharhinidae and most closely related to *C. falciformis* (Figure 1).

**Figure 1.**
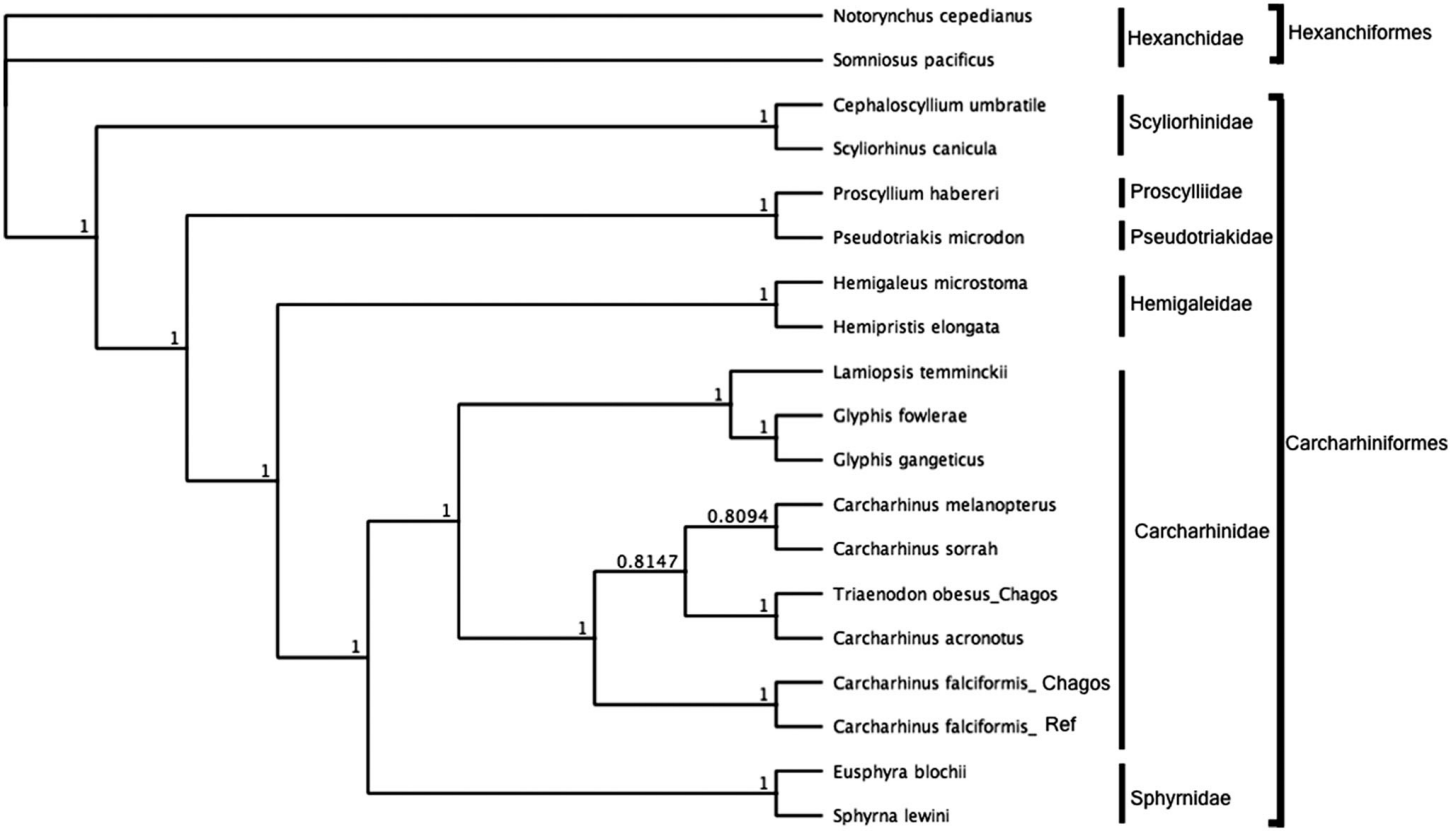
Bayesian estimate of phylogenetic position of *Carcharhinus falciformis* within the order Carcharhiniformes based on complete mitochondrial genomes. Members of the order Hexaniformes served as the outgroup. Families are indicated by vertical lines and orders by square brackets. Numbers at nodes are posterior probabilities. GenBank Accession Numbers: *Notorynchus cepedianus* (AB560489.1); *Sominosus pacificus* (AB560492.1)*; Cephaloscyllium umbratile* (KT003686.1); *Scyliorhinus canicula* (Y16067.1); *Proscyllium habereri* (KU721838.1); *Pseudotriakis microdon* (AB560493.1); *Hemipristis elongata* (KU508621.1); *Hemigaleus microstoma* (KT003687.1); *Lamiopsis temminckii* (KT698048.1); *Glyphis fowlerae* (KT698049.1); *G. gangeticus* (KT698040.1); *Carcharhinus melanopterus* (KJ720818.1); *C. sorrah* (KF612341.1); *C. falciformis_Ref* (MK092088); *C. falciformis_Chagos* (MN943498); *C. acronotus* (KF728380.1); *Triaenodon obesus*_*Chagos* (MN943497); *Eusphyra blochii* (KU892590.1); *Sphyrna lewini* (JX827259.1).

The mitochondrial genome of *C. falciformis* (GenBank: MN943498) was 16,701 bp in length and consisted of 13 protein-coding genes (PCGs), 22 tRNA genes, 2 rRNA genes, and a non-coding control region (D-loop). GC content was at 40.1%. All PCGs started with ATG and some PCGs ended with an incomplete stop codon. The control region was 1063 bp in length. Whole mitochondrial genome sequence of *C. falciformis* from the BIOT MPA will complement other studies of the species and will improve our understanding of distribution, abundance, catch and trade rates of the species' stock populations from the BIOT MPA. Overall, these studies will provide a measure of effectiveness and directions to improve conservation and management of marine populations in the BIOT MPA.

## Data Availability

Data which support the findings of this study are openly accessible in Genbank with the reference accession number MN943498.1 at DOI: https://www.ncbi.nlm.nih.gov/nuccore/MN943498.1.
